# Alternative High-Quality Hemolymph Extraction from Adult *Tenebrio molitor*: A Tool for Biotechnological and Immunological Research

**DOI:** 10.3390/insects17050499

**Published:** 2026-05-14

**Authors:** Mariela Alejandra Del Razo-Moreno, Rosa Estela Quiroz-Castañeda, Yazmín Alcalá-Canto, Hugo Aguilar-Díaz

**Affiliations:** 1 Centro Nacional de Investigación Disciplinaria en Salud Animal e Inocuidad, Instituto Nacional de Investigaciones Forestales, Agrícolas y Pecuarias, Carretera Federal Cuernavaca-Cuautla No. 8534, Progreso, Jiutepec 62550, Mexico; rammmvz@gmail.com (M.A.D.R.-M.); requiroz79@yahoo.com.mx (R.E.Q.-C.); 2 Departamento de Parasitología, Facultad de Medicina Veterinaria y Zootecnia, Universidad Nacional Autónoma de México, Av. Universidad 3000, Ciudad de Mexico 04510, Mexico

**Keywords:** hemolymph extraction, *Tenebrio molitor*, hemocytes

## Abstract

The mealworm beetle, *Tenebrio molitor*, is a coleopteran of importance for both immunological and biotechnological research, and it has even been considered as a potential nutraceutical. In recent years, the study of *T. molitor* has undergone significant development, including immune response, host–parasite interactions, and physiological approaches. However, to perform this type of study, one of the main obstacles is obtaining sufficient hemolymph and viable hemocytes. Thus, we developed a protocol for adult specimens that enables the collection of up to 300 μL of the hemolymph–anticoagulant buffer mixture per specimen, containing approximately 1.5 × 10^5^ hemocytes, with viability ranging from 85% to 90%. The technique involves a double mesothoracic puncture (DMP) and the use of a modified anticoagulant buffer that prevents hemolymph clotting, enabling continuous extravasation and ensuring high yields. Additionally, the hemocytes recovered with this protocol are intact and can be used for subsequent analysis. The hemolymph obtained using this protocol and its applications will help to better understand the processes involving hemolymph and its components in *T. molitor*, paving the way for further applications.

## Simple Summary

The mealworm beetle (*Tenebrio molitor*) is considered an insect with great biotechnological potential, especially for its uses as a nutraceutical and in environmental bioremediation. However, little is known about its molecular interactions with parasites and its immune system. For this reason, having a method that allows the extraction of hemolymph in significant quantities, with viable hemocytes, is a priority in the research on this beetle. Therefore, we present a protocol that allows the extraction of up to 300 µL of hemolymph containing hundreds of intact hemocytes with a viability of up to 90%. This contribution will undoubtedly enable greater progress in *T. molitor* research, thereby enhancing its application for the benefit of human and environmental health.

## 1. Introduction

Mealworms, *Tenebrio molitor*, are coleopterans that may serve as intermediate hosts for parasites affecting both animal and human health like *Hymenolepis nana*, the causative agent of human hymenolepiasis, which has an indirect lifecycle in which *T. molitor* is the intermediate host [1,2,3,4]. In biotechnology, *T. molitor* is considered a promising resource for nutraceuticals, given that processed larvae or powder have been approved by the European Union for use in food preparations [5,6]. This increased market demand requires a high-density *T. molitor* culture that meets the highest quality standards to achieve massive production in a healthy, pathogen-free environment [7,8]. Therefore, the creation of efficient protocols adaptable to automated processes for health monitoring and control are necessary. Therefore, the creation of efficient protocols adaptable to automated processes for health monitoring and control is necessary. In addition, under laboratory conditions, *T. molitor* participates in the biodegradation of polystyrene [9,10,11]. Thus, studying *T. molitor* tissues and organs is crucial for understanding its life cycle and the biological processes associated with it.

On the other hand, hemolymph plays a crucial role in an insect’s circulatory and immune system, serving as a source of nutrients, transporting molecules and hormones, and osmoregulation. This fluid, enriched with proteins, houses several types of cells, including hemocytes, which play a key role in protecting against pathogens [12]. So far, in *T. molitor*, the morphology of hemocytes has been characterized, identifying four main cell populations: prohemocytes, plasmatocytes, granule cells, and oenocytoids; however, the method for hemolymph extraction by coxae still yields a low volume [13,14]. Consequently, there is no optimized high-throughput method for extracting *T. molitor* hemolymph. In this respect, one of the main problems in obtaining hemolymph is coagulation and melanization, which occur rapidly in response to cuticle damage and immunological processes, including the activation of phenoloxidase (PO) [15]. It is important to note that other arthropod models of hemolymph extraction have reported that approximately 75% of hemocytes occur in circulation, while the other 25% are sessile and associated with tissues, which makes it difficult to access this cell population by conventional methods; however, this is something that still needs to be proven in *T. molitor* [16].

Furthermore, from a biotechnological perspective, hemolymph extraction and its study are necessary to comprehend cellular and immunological processes in *T. molitor*, including antimicrobial peptide isolation, host–vector–pathogen interactions, and environmental interactions [17,18,19].

Therefore, the present work describes a protocol based on a double mesothoracic puncture (DMP) and anticoagulant buffer injection for extracting hemolymph from *T. molitor.* This extraction allows for the isolation of high-quality hemolymph and high cell viability. In addition, this technique opens the possibility of accelerating progress in immunological, cellular, and molecular research on this beetle, which is of great importance to human health, food supplements, and biodegradation applications.

## 2. Materials and Methods

### 2.1. Experimental Design

In the protocol described here, each beetle was treated as its own unit of comparison (paired), so that the animal’s weight and size would not influence the outcome; animals of both sexes were also randomly selected. Then, 40 adult specimens aged 60 to 65 days post-hatching were used for the pairwise statistical analysis. The statistical design strategy involves using naturally paired data, i.e., two or more measurements taken on the same adult beetle. In our methodology, the reference method (Single Puncture in the Pro-mesothorax, SPP) was always performed first, followed by the DMP method for each specimen. Regarding the carry-over effect, hemolymph was collected continuously for 2 min under anesthesia. Because our method employs an anticoagulant buffer, it prevents tissue or debris from clumping.

### 2.2. Biological Sample Preparation

The *T. molitor* strain INIFAP CENID-SAI Conventional was used, maintained, and expanded under standard laboratory conditions in open colonies with basic biosafety systems. The health status of the population was monitored periodically, confirming the absence of clinical signs of disease or evident parasitosis. A colony of adult *T. molitor* beetles (equal proportion of males and females) was maintained on a substrate composed of 35% oat flour (Granvita^®^, Grupo Industrial Vida S.A. de C.V., Zapopan, Mexico), 60% whole wheat flour (Tres Estrellas^®^, Harinera Los Pirineos, S.A. de C.V., Salamanca, Mexico), and 5% whole oat flakes (Quaker^®^, Ciudad de Mexico, Mexico) at 28 °C with 12 h light/dark photoperiods, with water and food *ad libitum*. The *T. molitor* specimens were classified into small, medium, and large larvae, pupae, and adults in different containers (32 cm length × 19 cm width × 10.5 cm depth). The substrate composition is basic for the nutrition and well-being of the specimens. The flour mixture provides nutrients, and the addition of whole oat flakes creates an environment that facilitates natural burrowing and moving behaviors, reducing the stress associated with confinement. To maintain hygiene, the substrate was changed every 15 days, and vegetables were added twice a week to ensure a constant supply of fresh food and moisture. With this diet, the larval life cycle lasted approximately 8–12 weeks after hatching, after which they entered the pupation stage. For this experiment, adult beetles (60–65 days after pupal emergence) were used. As the age of the beetle may affect the yield and quality of the hemolymph, we did not use senescent specimens because they may have a stiffer cuticle and affect the hemolymph yield. Before extraction, we separated adult beetles (with an average length of 2 cm and a width of 0.5–0.6 cm) and fasted them for 72 h with sterile water *ad libitum* to clear gut waste. We anesthetized the beetles as follows: each specimen was placed in a 100 mm Petri dish (Corning™, Corning, NY, USA) with a cotton swab soaked in 0.3–0.5 mL of isoflurane (PiSA Agropecuaria, Guadalajara, Mexico), sealed with plastic film (Parafilm^®^, Sigma-Aldrich, London, UK), and incubated at room temperature for 25 min [20]. Then, we incubated the beetles at −20 °C for 10 min and checked for appendage movement to confirm the anesthetic effect.

Following anesthesia, beetles were washed twice by immersion in cold 10% benzalkonium chloride (Meyer, Labs Mexico, Mexicali, Mexico) for 10 min. We discarded the excess and rinsed the beetles three times by immersion/agitation in cold sterile distilled water for 2 min. This was followed by a wash with 1 mL of cold antibiotic–antimycotic mixture (1:100) (10,000 units/mL penicillin, 10,000 μg/mL streptomycin, and 25 μg/mL amphotericin B) (Thermo Fisher Scientific, Waltham, MA, USA) in distilled water for 10 min. Finally, we rinsed the specimens twice with 1 mL of cold, sterile distilled water, then dried them on sterile gauze [15]. Cold conditions are crucial for optimizing the quantity and quality of extracted hemolymph, thereby preserving the integrity and viability of hemocytes [15].

Before hemolymph extraction, we centrifuged the whole specimens (200× *g* for 1 min at 4 °C) in a conical tube with sterile distilled water to significantly reduce contaminants.

### 2.3. Hemolymph Extraction

**Figure 1:**
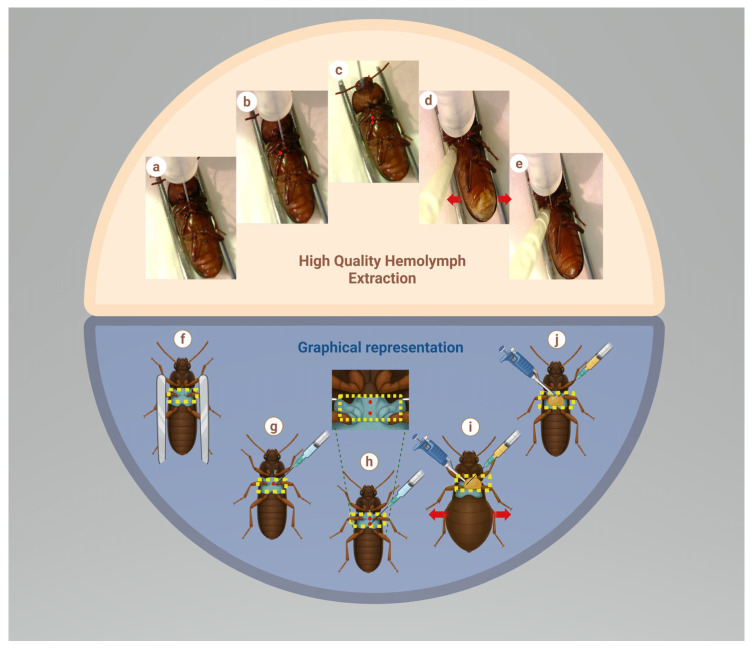
Steps of the hemolymph extraction from adult *T. molitor* by DMP and anticoagulant buffer injection. (**a**) The beetle is positioned ventrally with dissecting forceps. (**b**) A first puncture is performed in the ventral region of the anterior mesothorax. (**c**) A second puncture is performed 1 mm below the first (this contributes to hemolymph extravasation by intracoelomic pressure). (**d**) Anticoagulant buffer is injected into the beetle’s hemocoel (an abdominal bulge should be observed, indicated by red arrows). (**e**) Sterile hemolymph mixed with anticoagulant buffer is collected through pipetting. (**f**–**j**) Graphical representation of the hemolymph extraction by the DMP method.

Specimens were held laterally with sterile flat-tip forceps and positioned ventrally under a stereomicroscope. We performed two punctures in the ventral region of the anterior mesothorax, near the promesothoracic joint. The first puncture was performed at a 45° angle to the beetle’s body plane. Immediately afterwards, a second puncture was performed 1 mm below the first, maintaining a 45° angle. We slowly injected 300 μL of cold anticoagulant buffer with a 31 G × 6 mm, 0.5 mL Ultra-Fine syringe (Becton, Dickinson and Company, Franklin Lakes, NJ, USA). At the same time, we collected the extravasated hemolymph–anticoagulant buffer mixture (Figure 1) and transferred it to a sterile 1.5 mL microtube. This was gently mixed by pipetting and stored at 4 °C until it was ready for use.

The buffer must be administered completely to avoid increasing the insect’s normal intracoelomic pressure [21]. An abdominal bulge indicates the correct insertion of the buffer. To maintain sterile conditions during hemolymph extraction, we recommend using a sterile laminar-flow hood or Fisher burners. The first puncture provides a controlled flow system that reduces pressure and improves hemolymph extravasation, as excessive pressure could rupture the beetle’s internal tissues.

The anticoagulant buffer (AB) preparation is as follows: Dissolve one tablet of Phosphate-Buffered Saline (PBS) (Sigma, P4417, St. Louis, MO, USA) in 200 mL of sterile distilled water. Add 4.16 g of anhydrous dextrose (Fermont, Lot 08401, Atlantic City, NJ, USA) and 1.6 g of dehydrated sodium citrate (Sigma, S-4641, USA), and verify pH to 7.4. Sterilize by filtering and store at 4 °C for up to 3 months.

### 2.4. Hemocyte Separation

We centrifuged the collected hemolymph at 300× *g* for 4 min at 4 °C. We decanted the supernatant and washed the hemocyte pellet twice through centrifugation using the anticoagulant buffer as previously described. We gently resuspended the hemocyte pellet by pipetting in 100 μL of cold sterile anticoagulant buffer. It is essential to note that purified hemolymph should be clear and colorless; turbidity may indicate contamination with cellular debris or melanin.

All hemolymph extractions from SPP used as controls were performed as described by [13]. Briefly, beetles were anesthetized in a cold chamber at 4 °C for three minutes. The hemolymph was collected by using a 29-gauge needle at the ventral level of the pro-mesothorax articulation and mixed with 3 μL of phosphate buffer (PBS, 10 mM pH 7.4; Merck Life Science, Milan, Italy).

### 2.5. Hemocyte Quantification

We transferred 10 μL of the obtained hemocytes into a sterile microtube and diluted at 1:2 with a 4% trypan blue solution in cold anticoagulant buffer at 4 °C (Thermo Fisher Scientific, Waltham, MA, USA). This was mixed gently by pipetting. We loaded 10 μL of the mixture (hemocytes/anticoagulant buffer/trypan blue) into a Neubauer chamber and observed them under a conventional light microscope (Motic B1-220A, Xiamen, China). We counted the number of cells in the four corner quadrants. The total number of hemocytes was calculated by considering the following formula [16]:

Hemocyte concentration (cell/mL) = (Total cells counted/Number of squares counted) × dilution factor × 10^4^

If it is available, the use of an automatic cell counter (CytoSmart Cell Counter, Corning™, Corning, NY, USA) can improve cell counting accuracy and viability.

### 2.6. Hemocyte Giemsa Staining

We centrifuged the extracted hemolymph at 300× *g* for 4 min at 4 °C. The supernatant was discarded. We resuspended the cell pellet in 50 µL of PBS and placed 20 µL on a contaminant-free slide. A smear was taken, ensuring even coverage of a slide. We dried the sample for 5 min at room temperature. The sample was fixed by dripping absolute methanol (J.T. Baker, Seattle, WA, USA) onto the entire smear and incubating for 5 min. We dried the sample at room temperature, then immediately passed the slide over a flame three times, gently, using a Bunsen burner.

**Figure 2:**
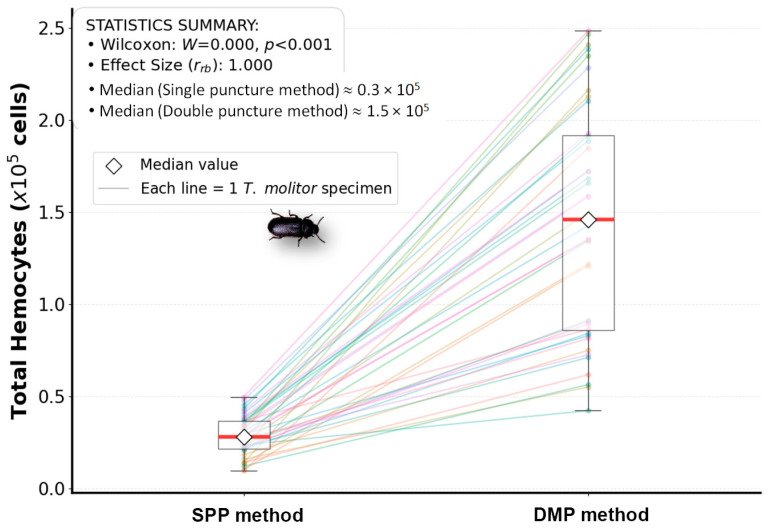
Comparative paired statistical analysis of hemocyte yield in *T. molitor* (*n* = 40) using the reference method SPP and the DMP method. The Raincloud plot combines density curves, representing an empirical distribution. Box plots show the median, and individual lines illustrate the relationship between the two measurements for each specimen (each line correspond to a specimen). An upward trend is observed in all cases. (Performed with Anaconda Toolbox Python Projects v4.20.0). The Wilcoxon signed rank test yielded W = 0.000 and a *p* < 0.001. The effect size was 1.000 (95% bootstrap CI: 1.000–1.000; 10,000 resamples), with all 40/40 specimens showing higher total hemocyte count with DMP.

The sample was stained with pre-filtered Giemsa solution (MERCK, Darmstadt, Germany) diluted 1:10 in PBS, covering completely the smear and incubating for 25 min at room temperature. We placed the slide upright and gently rinsed it three times with distilled water. We add a few drops of mounting medium (MERCK, Germany) to the smear, then placed a clean coverslip on top. We sealed the edges with conventional varnish, and then observed them under a bright-field microscope (Figure 2).

### 2.7. Quantification and Fluorescent Vital Staining of Hemocytes

Hemocytes were counted as described above. Cellular viability and metabolic activities were assessed using 5(6)-carboxyfluorescein diacetate (CFDA, Thermo Fisher Scientific, Waltham, MA, USA). Briefly, we resuspended 1 × 10^5^ hemocytes in 100 μL of room temperature anticoagulant buffer and added 1 μg/μL of CFDA solution (stock solution: 10 μM CFDA in DMSO). This was resuspended and incubated at 26 °C for 20 min with gentle agitation (Benchmark Scientific IncuShaker, Sayreville, NJ, USA). We centrifuged the solution at 300× *g* for 4 min, washed the pellet, and then resuspended it in anticoagulant buffer and incubated it for 20 min in darkness at 26 °C. Observations were conducted using a wide-field fluorescence microscope (Zeiss Axioskop 40 HBO 50, Jena, Germany) with a Hg lamp and a 100× FITC filter. The experiment was conducted in three replicates, with three repetitions per sample.

### 2.8. Statistical Analysis

A Wilcoxon signed-rank test (W) is a nonparametric statistical test that examines differences between two paired samples, and it was used to compare hemolymph extraction yields between the SPP and the DMP methods. All analyses were conducted using JASP software (version 0.16.3).

## 3. Results

### 3.1. Hemolymph Yield and Extraction Efficiency

The *T. molitor* hemolymph was extracted using DMP combined with the injection of an anticoagulant buffer (Figure 1). The protocol described in this work yields approximately 300 μL of hemolymph–anticoagulant buffer mixture per specimen, resulting in approximately 1.5 × 10^5^ hemocytes with 85–90% viability (Table 1; Video S1). A comparison between the protocol described in this work and other methods is presented in Table 2.

### 3.2. Statistical Analysis of Hemocyte Count

Differences were evaluated as paired groups. The Shapiro–Wilk test was used, revealing that the data from our method (DMP) do not follow a normal distribution (W = 0.942, *p* = 0.041), justifying the use of a nonparametric test such as the Wilcoxon signed-rank test (JASP v0.16.3). The optimized method (DMP) yielded higher cell recovery, with a median of 146.19 × 10^3^, corresponding to approximately 1.5 × 10^5^ hemocytes. In contrast, the SPP method resulted in a median of 28.09 × 10^3^ hemocytes, equivalent to approximately 0.3 × 10^5^. Thus, the DMP method increased recovery by at least ~94.862 × 10^3^ hemocytes per beetle, as estimated by the Hodges–Lehmann estimator (95% CI), (Figure 2). This finding is consistent with the approximately fivefold increase observed relative to the SPP method.

### 3.3. Hemocytes Characterization

**Figure 3:**
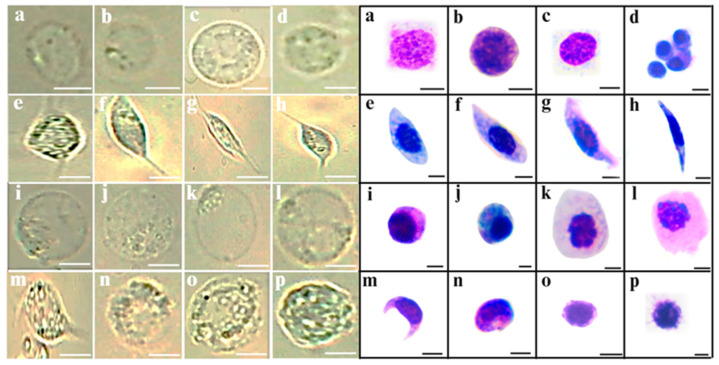
Morphological characterization of *T. molitor* hemocytes purified from hemolymph extracted by DMP. (**Left panel**): conventional light microscopy images (phase contrast). (**a**–**d**) Prohemocytes (5–8 μm); (**e**–**h**) plasmatocytes (9–15 μm); (**i**–**l**) oenocytoids (12–15 μm); (**m**–**p**) granulocytes (8–11 μm). (**Right panel**): hemolymph smear Giemsa staining. (**a**–**d**) Prohemocytes (basophilic cytoplasm, 5–8 μm, large nucleus); (**e**–**h**) plasmatocytes (elongated, 9–15 μm, central oval nucleus); (**i**–**l**) oenocytoids (eosinophilic cytoplasm, 12–15 μm, eccentric nucleus); (**m**–**p**) granulocytes (abundant metachromatic cytoplasmic granules, 8–11 μm). Scale bar, 5 μm.

The quality of the hemocytes recovered after hemolymph extraction was assessed using Giemsa staining. This staining revealed the variety of hemocyte types, including prohemocytes, plasmatocytes, and oenocytoids, among others, as has been reported by [13], which described them based on their morphological features and staining affinity. Microscopy visualization and Giemsa staining revealed the cells’ integrity and enabled the characterization of hemocyte types (Figure 3).

### 3.4. Hemocytes Viability

**Figure 4:**
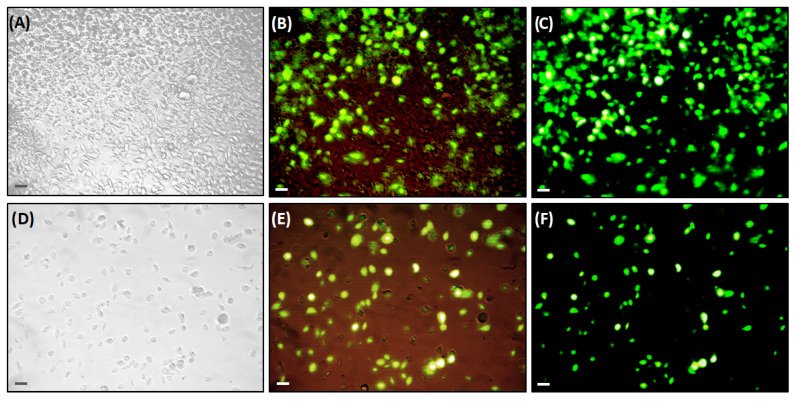
CFDA staining to assess the quality and viability of hemocytes obtained from hemolymph extracted using the DMP method after 90 min (**A**–**C**) and 5 h (**D**–**F**) at room temperature in anticoagulant buffer. (**A**) Bright field micrograph (light microscopy) of intact and hemocytes with a viability of 90% (40×). (**B**) Bright field and CFDA staining (green fluorescence) merge micrographs of hemocytes with 90% viability. (**C**) Confocal micrograph (fluorescence microscopy) of hemocytes stained with CFDA (green fluorescence) and 90% viability. (**D**) Bright field micrograph (light microscopy) of hemocytes after 5 h, with 30% viability (40×). (**E**) Bright field and CFDA staining (green fluorescence) merge micrographs of hemocytes with 30% viability. (**F**) Confocal micrograph (fluorescence microscopy) of hemocytes stained with CFDA (green fluorescence) and 30% viability. Fluorescent hemocytes indicate viable cells (100×). Scale bar is 20 μm. The micrographs are representative of and were conducted in three replicates, with three repetitions per sample.

*T. molitor* hemocytes stained with CFDA revealed the integrity and viability of these cells and the potential to be used in further experiments. The viability of the hemocytes was 90% after 90 min at room temperature, and decreased to 30% after 5 h at the same temperature in anticoagulant buffer (Figure 4; Table 3).

## 4. Discussion

The study of the mealworm beetle, *T. molitor,* has gained interest due to its numerous applications, including the degradation of EPS, its nutraceutical potential [9,29], and its use as a model for various immunological studies in the vector–pathogen relationship [30].

The use of insects for human consumption demands strict quality standards, which depend directly on maintaining a pathogen-free colony, a requirement for the insect’s robust immune response [8]. Therefore, implementing a hemolymph extraction protocol for immune analysis could contribute to the overall health and well-being of the production. To date, despite its importance, the methods for extracting hemolymph from *T. molitor* have been limited, resulting in low cell viability and poor yields. Thus, this work presents a high-quality, high-yield method for extracting *T. molitor* hemolymph with contaminant-free hemocytes. In this regard, the results showed a yield of approximately 300 μL of a hemolymph–anticoagulant buffer mixture per specimen, yielding approximately 1.5 × 10^5^ hemocytes with 85–90% viability. This represents a 5-fold increase over the hemocytes obtained with the SPP method, in which we obtained 3 × 10^4^ hemocytes in 3 μL of hemolymph [13]. The statistical analyses using the Wilcoxon signed-rank test revealed significant differences (W = 0.000, *p* < 0.001), consistently favoring DMP over the SPP method.

On the other hand, in this protocol, we also highlight the use of isoflurane as an anesthetic agent, which was critical for maintaining cellular viability. In contrast to reports of traditional anesthesia methods, isoflurane has not shown negative cellular effects, at least as reported in *Drosophila* and honey bee [25,31]. It is worth noting that anesthesia by freezing results in extensive cellular damage due to the formation of ice crystals [20,32,33]. Moreover, other anesthetics, such as CO_2_ and ethanol, cause acidification of hemolymph and retraction and mucus secretion, respectively; both compromising hemolymph quality [23,34].

One critical step in our protocol involves a first puncture in the beetle mesothorax to avoid intracoelomic pressure, followed by a second puncture to induce hemolymph extravasation via buffer injection. Both punctures generate a controlled buffer efflux to avoid any damage to internal tissues [15,35]. In arthropods such as mosquitoes, approximately 75% of hemocytes circulate, while the remaining 25% are sessile and associated with tissues [16]. According to this, if a similar hemocyte distribution exists in *T. molitor*, our buffer injection could trigger the necessary efflux to mobilize both circulating and tissue-associated hemocytes, allowing the isolation of different populations with viability greater than 90%. Regarding hemolymph quality, the samples obtained were clear and colorless, as a cloudy color suggests contamination. Because we performed a pre-extraction wash with benzalkonium chloride and the antibiotic–antimycotic mixture to remove surface contaminants, we obtained a clear hemolymph sample. It is also important to consider factors that could directly influence extraction efficiency and reduce contamination, such as the age and hydration level of the insect, as well as solid starvation for up to 72 h [36,37].

In *T. molitor* and larger tenebrionids, such as *Zophobas morio*, leg-cutting can recover up to 15 µL and 30 µL per specimen, respectively [28]. Therefore, we may conclude that the DMP method achieves excellent yields, rivaling those of larger organisms. Furthermore, by adapting our method to larger beetles, cell yields could be even better.

Here, we report a protocol that yields more hemolymph and provides better cell viability and quality. We believe that the anticoagulant buffer composition significantly diminishes hemolymph coagulation and provides a carbon source to the cells. Additionally, it induces an intracoelomic flow in the beetle, favoring a significant recovery.

## 5. Conclusions

The methodology presented in this work for collecting high-quality hemolymph significantly enhances hemocyte recovery in *T. molitor*, enabling researchers to use fewer specimens, reduce costs, and minimize colony maintenance time. Notably, increased hemocyte yield is especially valuable for studies where biological materials are typically scarce. Additionally, the potential applications of this protocol include vector–pathogenic interactions, the immune system, molecular approaches, and microbiome studies, all of which contribute to beetle biology elucidation.

## Figures and Tables

**Table 1:** Statistical analysis of the number of hemocytes and volume of hemolymph recovered in DMP and SPP methods. Lower values (<10%) of the coefficient of variation indicate lower variability and consistency. N, specimens; SD, standard deviation.

Extraction Method/Variable	N	Mean	SD	Coefficient of Variation (%)
DMP method (No. hemocytes)	40	146,190	8663	0.059 (5.90%)
DMP method hemolymph recovery (µL)	40	266,820	14,948	0.056 (5.6%)
SPP method (No. hemocytes)	40	28,090	4623	0.1640 (16.40%)
SPP method hemolymph recovery (µL)	40	2.8	0.5	0.1785 (17.85%)

**Table 2:** Comparison of major features of the protocol based on DMP and other reported methods.

	Other Methods	Advantages of New Protocol
Anesthetic effects	CO_2_ causes hemolymph acidification [22] Ethanol 70% induces excessive mucus secretion, affecting hemolymph quality [23] Ethyl acetate, usually stuns insects quickly but kills them slowly; specimens, even though they appear dead, may revive if removed from the killing jars too soon [24] Cold (4 °C) induces temporal immobilization [25]	Isoflurane is considered a benign alternative [25]
Hemolymph yield	Method: Coxae cut, 2 μL [26] Punction in ventral pro-mesothorax articulation, 3 μL [27] Cutting the tibia of the first pair of legs, 15 μL [28]	300 μL of a hemolymph–anticoagulant buffer mixture per specimen
Hemocytes yield and viability	Method: Coxae cut, not evaluated [26] Punction in pro-mesothorax articulation method, 3 × 10^4^ hemocytes [27] Cutting the tibia of the first pair of legs, not reported [28]	1.5 × 10^5^ hemocytes with 85–90% viability (CFDA)
Applications	Method: Coxae cut; counting and apoptosis studies [26] Punction in ventral pro-mesothorax articulation; phagocytosis and total hemocyte count [27] Cutting the tibia of the first pair of legs; biochemical properties of hemolymph studies [28]	Cellular and immunological assays with viable hemocytes

**Table 3:** Statistical analysis of the number of hemocytes recovered using the DMP method. The total and viable numbers of hemocytes were calculated, along with their coefficient of variation and the percentage of viability. SD, standard deviation.

Time	Mean Total Hemocytes	SD	Coefficient of Variation (%)	Mean Viable Hemocytes	SD	Coefficient of Variation (%)	Viability Percentage (%)
90 min	148,796	7895	0.053 (5.3%)	133,916	8231	0.061 (6.1%)	90%
5 h	136,520	8486	0.062 (6.2%)	40,956	2957	0.072 (7.2%)	30%

## Data Availability

The raw data supporting the conclusions of this article will be made available by the authors on request.

## Supplementary Materials

The following supporting information can be downloaded at: https://www.mdpi.com/article/10.3390/insects17050499/s1, Video S1: *Tenebrio molitor* hemolymph extraction protocol.
